# Qualitative interviews to understand methods and systems used to collect ethnicity information in health administrative data sources in England

**DOI:** 10.12688/wellcomeopenres.19262.1

**Published:** 2023-06-21

**Authors:** Gemma Quayle, Bethan Jones, Jessica Atkins, Caitriona Shannon, Roxanne Smith, David Tabor, Zuzanna Bałabuch, Courtney Cox, Sarah Horsell, Marie John, Tomas McGrail White, Sophie Vickers, Sophia Whittinger, Neil Bannister, Veena Raleigh, Bilal Mateen, Rosemary Drummond

**Affiliations:** 1 Office for National Statistics, Newport, NP10 8XG, UK; 2 The King's Fund, London, England, W1G 0AN, UK; 3 Nuffield Trust, London, England, UK; 4 Wellcome Trust, London, England, NW1 2BE, UK

**Keywords:** Ethnicity, race, health disparities, health inequality, ethnic minority, data quality

## Abstract

**Background:** This article is one of a series aiming to inform analytical methods to improve comparability of estimates of ethnic health disparities based on different sources. This article explores the quality of ethnicity data and identifies potential sources of bias when ethnicity information is collected in three key NHS data sources. Future research can build on these findings to explore analytical methods to mitigate biases.

**Methods:** Thematic analysis of semi-structured qualitative interviews to explore potential sources of error and bias in the process of collecting ethnicity information across three NHS data sources: General Practice Extraction Service (GPES) Data for Pandemic Planning and Research (GDPPR), Hospital Episode Statistics (HES) and Improving Access to Psychological Therapies (IAPT). The study included feedback from 22 experts working on different aspects of health admin data collection for England (including staff from NHS Digital, IT system suppliers and relevant healthcare service providers).

**Results:** Potential sources of error and bias were identified across data collection, data processing and quality assurance processes. Similar issues were identified for all three sources. Our analysis revealed three main themes which can result in bias and inaccuracies in ethnicity data recorded: data infrastructure challenges, human challenges, and institutional challenges.

**Conclusions:** Findings highlighted that analysts using health admin data should be aware of the main sources of potential error and bias in health admin data, and be mindful that the main sources of error identified are more likely to affect the ethnicity data for ethnic minority groups. Where possible, analysts should describe and seek to account for this bias in their research.

## Introduction

### Project background

There is significant public and government interest in addressing health inequalities. During the coronavirus (COVID-19) pandemic, higher mortality rates among some ethnic minority groups were quickly identified and it became even more urgent to improve the evidence base around ethnicity and health. Existing literature highlights issues with the quality of ethnicity information collected in the NHS. Bias caused by inconsistencies in the quality and completeness of ethnicity information recorded in the different data sources can affect the validity of analyses using each dataset. This bias can also make it difficult to draw meaningful comparisons between studies based on different sources.

Although inconsistencies in the quality of ethnicity data between different sources can make comparisons more challenging, there are also opportunities to improve data quality and completeness through data linkage. For example, NHS Digital have created an Ethnic Category Information Asset that amalgamates ethnicity information from GP and hospital records to help improve coverage.

Although statistical analysis was not the initial purpose of these administrative data sources, and is not their primary function, these types of administrative data are now being used more regularly to create statistics. Administrative health data can be used for statistical analysis successfully but to do so there is a need to understand both their benefits and limitations.

This desk review explores potential sources of error and bias in the process of collecting NHS ethnicity information. It is part of a wider research collaboration between ONS, Wellcome and the Race Equality Foundation to improve understanding of the quality of ethnicity data in key NHS sources, and develop methodological solutions to mitigate biases. To complement the findings from this desk review, the wider research project also includes:

- Quantitative person-level comparisons of ethnicity information recorded in key health data sources, compared to ethnicity information from the Census (which is widely regarded as the most robust ethnicity data source covering the whole population as unlike other sources we can be confident Census ethnicity is self-reported).
- Focus groups to provide further insights from the public and healthcare staff on their experiences of collecting ethnicity information data in practice, and potential for errors or bias to be introduced.

### Research aims and approach

This research aims to:

- Explore and describe potential sources of bias and error in the process of collecting and processing ethnicity data from key health data sources.
- Use this information to draw conclusions about the reliability of the different data sources and the main sources of bias, outlining what implications this has for analysts.

Future research will build on these findings to propose methods analysts can use to mitigate biases when analysing ethnicity information from health admin data.

## Methods

### Background information on the data sources in scope

When patients interact with different NHS services, administrative data is collected to facilitate effective service delivery and monitoring. Some of these data are compiled and made available for secondary, non-clinical uses (such as healthcare planning, to reimburse healthcare providers for their services and for research).

Three NHS administrative data sources in England were included in the analysis: General Practice Extraction Service Data for Pandemic Planning and Research (GPES, GDPPR), Hospital Episode Statistics (HES) and Improving Access to Psychological Therapies (IAPT). These sources cover both primary and secondary care, and the Office for National Statistics (ONS) has previously published person-level comparisons of the ethnicity data recorded in these sources compared with 2011 Census ethnicity information.

The sources of error and bias affecting these three sources provide insight into the types of issues that could be relevant to other electronic administrative health records. However, these three sources are not intended to be representative of issues affecting all health data sources.

***Ethics***

Prior to the research, a data ethics self-assessment was completed using the ONS self-assessment template. This is a process overseen by the National Statistician’s Data Ethics Advisory Committee. The risk scored in the self-assessment was lower than the threshold for which further ethics approval is required by the Committee.

***Consent***

Consent to record and use anonymised quotes was obtained from participants prior to interview. Participants had the right to withdraw either during, or after the interview, up until the point of publication. Participants were given our contact details and, had they wished to withdraw their data within the aforementioned time frame, their serial number would be matched to their data set and the data would be removed and deleted. All data is anonymised, kept confidential and securely stored. Participants are not identifiable in published outputs. Data was and will be securely held on a restricted SharePoint database where only the research team have access, until the point of deletion.

### Expert interviews

***Sampling and recruitment***

Participants were recruited purposively and through snowball sampling measures. Participants were selected for their knowledge and experience of working with the data source under investigation. Between January and March 2022, a total of 21 participants were interviewed, and one additional participant provided comment through email. Five participants worked with the GDPPR data source, eight participants worked with the IAPT data source (one of whom provided email information only) and nine participants worked with the HES data source. Participants worked in a variety of roles and across a variety of employers including NHS England, NHS Digital, service providers and IT systems suppliers.

Staff from service providers in data processing roles or managerial roles (e.g. GP Practice Manager) were in scope of the recruitment, but staff who routinely collect ethnicity information from patients were excluded. This decision was to avoid duplication with a separate study as part of the wider research project which involved focus groups to provide further insights from the public and healthcare staff on their experiences of collecting ethnicity information data in practice.

***Data collection***

A desk review (including published and grey literature) was conducted for each data source to inform the research questions for interviews.

Semi-structured interviews were the selected data collection method to ensure the research questions were addressed, while also providing flexibility for participants to express their experiences in detail. 

Researchers designed and peer-reviewed topic guides which were informed by the literature review and were piloted.

Interviews were conducted by a lead interviewer on Microsoft Teams and lasted between 40 to 80 minutes. A second researcher attended the interview as an observer to take notes of responses. Interviews were recorded and digitally transcribed using the Microsoft Teams transcription function. All data were held in secure SharePoint folders accessible only to the research team involved.

***Analysis***

Recordings, transcriptions, and observer notes formed the data generated from the semi-structured interviews. All data collected was anonymised by removing names and identifiable details from the transcripts. Transcripts of the interviews were reviewed against their respective recordings as quality assurance.

A framework was created which followed the general structure of the topic guide, from which transcripts were sorted, and coded. Thematic analysis took place in a session with all interviewers and observers, where observer notes were reviewed, emerging themes were discussed and recorded, and interviewer reflection took place. Further analysis was then conducted using the organised data within the framework to explore emerging themes across interviews and produce high-level findings.

Reflexivity was facilitated and encouraged across all researchers with the view to keep researcher influence to a minimum. Data collection, analysis and reporting was a collaborative process involving many researchers so that the influence of individual characteristics was reflected upon and reduced.

## Results

We begin with a summary of the main findings, before exploring the main themes in more detail.

### Summary of main findings

This summary highlights how ethnicity data are collected from a patient, and move through a process of data collection, processing, and quality assurance across all three data sources to prepare the data for analysis. Potential sources of error and bias identified throughout this process, and across all three data sources, are also summarised.

***Summary of potential sources of error and bias throughout data collection process***

**Figure 1.**
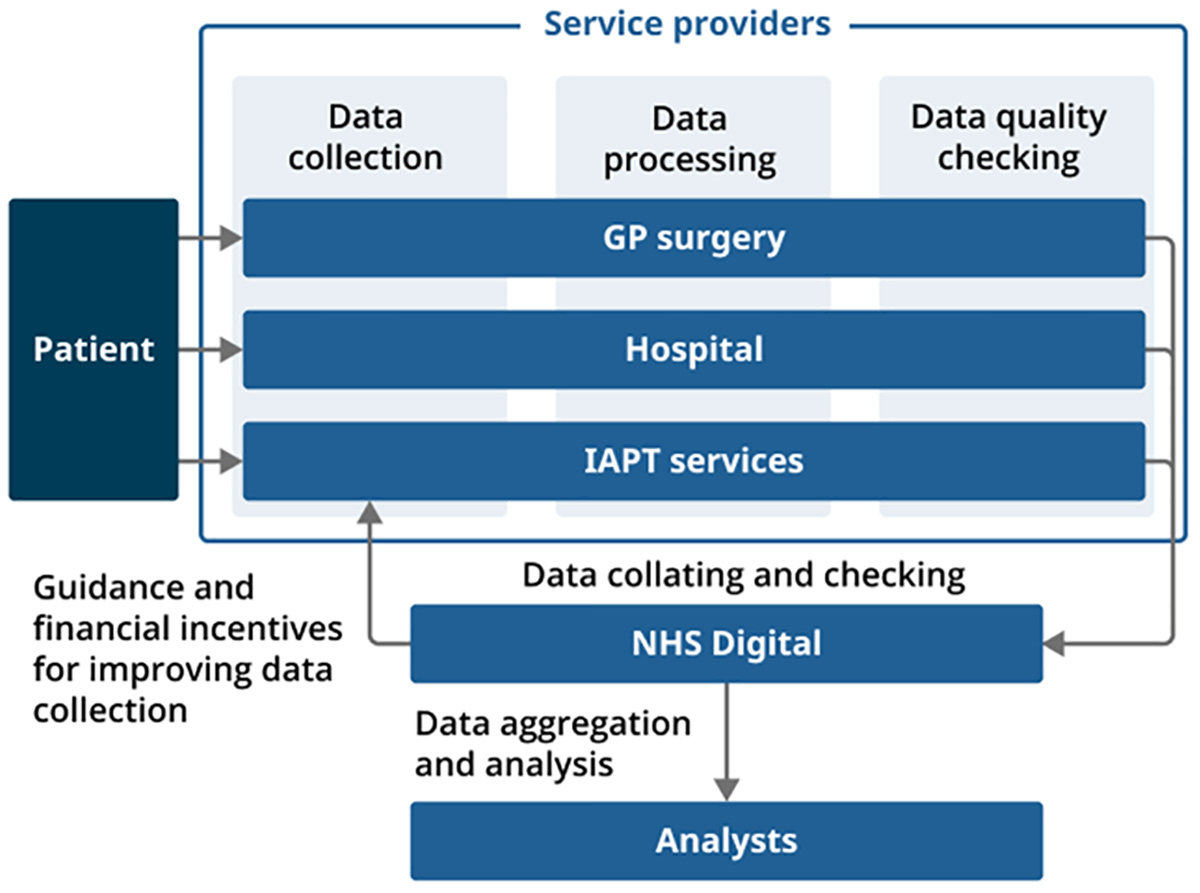
Summary of data collection process for three health data sources in England. Source: Office for National Statistics - Methods and systems used to collect ethnicity information in health administrative data sources, England: 2022.

Our research identified ways in which data quality could be affected at different stages of the data collection process shown in Figure 1. These are summarised as follows.

Patient

For some sources, patients can opt out of data sharing, leading to missingness in data for analysis.

Service providers

Data are collected by each healthcare provider (GP surgeries, hospital departments and Improving Access to Psychological Therapies (IAPT) service providers). For all three data sources, participants described multiple methods for collecting ethnicity data, some of which are self-completion (for example, an online or paper form) and some are completed by a third party (for example, staff asking patients face-to-face or on the phone). Service providers also carry out some data processing and quality checks.

1.   Data collection

- Variation in detailed ethnicity categories collected locally.
- Different data collection modes.
- Inconsistent use of residual categories: “Not stated”, “Not known”.
- Staff understanding of value of ethnicity data varied.
- Different staff may collect data slightly differently.
- Same data collected multiple times; little known about reliability of different entries.

2.   Data processing

- Mapping detailed ethnicity categories to aggregate harmonised categories loses granularity.
- Complex and subjective coding processes for some data sources.

3.   Quality checks

- Trade-off between high-quality data and burden on service providers.
- Staff understanding and implementation of quality checks varied.

NHS Digital

- NHS Digital quality checks focus on completeness; checking accuracy is more challenging.
- Guidance on data collection and data processing limited for some sources
- Any financial incentives focus on completeness.

### Detailed findings

This section presents more detailed findings from our research presented under the following themes: Data Collection, Data Processing, Quality Checks, Training and Guidance, Patient Opt Out from Data Sharing.

***Data collection***

Transfer of existing health records for data collection varied between sources

Ethnicity information is collected at the first point of contact or when registering with a specific service provider.

The extent to which existing health records were used varied between the data sources. The use of existing health records depended on whether previously collected data was available on local systems and easily transferable to the patient’s current service provider.

Different data collection systems mean that, for all three sources, a patient could have their ethnicity recorded multiple times and “ *they may have different ethnic categories submitted*” each time. It was highlighted *“if you've got different ethnicities for the same patient you don’t know which is the actual one 'cause you don't have a single source of truth.”* No evidence was found of checks to confirm why different categories were submitted or if self-reported ethnicity had changed.

Variation in ethnicity categories collected locally

NHS Digital specifies data standards for ethnicity data, including a standardised list of ethnicity categories ( NHS data dictionary), which are based on the 16+1 categories defined in the 2001 Census. This NHS Data Model & Dictionary is issued to both providers and their system suppliers to specify how the data should be captured. While participants were aware of these standards, they implied a sense of flexibility at the local level of ways to reach these standards, and that service providers *“can do whatever they want locally”.*

For all three sources, we found evidence of more detailed ethnicity categories used at a local level for data collection and analysis than those required by NHS Digital. Participants implied the reason for this is because the Census categories were not meeting needs locally:

*“we locally record the ethnicity a lot more granular than what we submit nationally… the national data isn’t as useful for local demographics as…the local data is.”*

These detailed categories are not consistent between service providers and IT systems, even for the same data source. This has important implications for the quality of data collected, as differences in how the ethnicity question is asked, and which ethnicity categories are presented, could influence an individual’s response. Furthermore, the use of bespoke categories has implications later in the process when they are mapped to Census categories for national level analyses (see Data Processing section).

The mode of data collection could impact data completeness

For all three data sources, participants described multiple modes for collecting ethnicity data. This can include both the format of data collection; whether ethnicity data is captured online or on a paper form, and who records the information; whether the form is self-completed by a patient or whether a member of staff records the patient’s response on their behalf. 

For GP and IAPT, participants did not flag variation in data collection methods as a major source of error or bias, though there was acknowledgement that unlike paper forms, online forms can allow warning messages to encourage completion of ethnicity information which could improve completeness.

Participants also mentioned how the COVID-19 pandemic influenced the use of different data collection modes, and that this could have a corresponding impact on data completeness. For GP data, participants mentioned that the process of registering in person during the COVID-19 pandemic was less patient-friendly, and services offered on registration were no longer available.

*“so we're we are seeing that you know people aren't…being recorded because we haven't been able to offer the new patient check like we used to because of COVID”*

For HES, participants reported that they expected less complete ethnicity reporting during the pandemic, as during telephone consultations clinical information may have been prioritised over demographic variables.

Variation in how the question is presented for different modes could impact accuracy

For GP and IAPT, participants indicated the question and ethnicity categories are generally consistent across online and paper forms. However for HES, the likelihood of patients being shown identical lists of ethnicity categories, or any list at all, appeared to vary by mode of data collection and department. Although each hospital department “ *should have a card which describes all the different ethnicities”* which is “ *shared with the patient*”, it was not clear how consistently this was implemented.

Role of staff in data collection could impact accuracy

For HES and GP data, concerns were raised around the impact of staff collecting and recording data, and whether staff had adequate training to ensure data quality.

For example, participants reported that staff inputting patients’ responses at GP surgeries created opportunities for bias through subjective interpretation, if the response provided by the patient did not exactly match the ethnicity category options available. There was also evidence of ethnicity being conflated with country of birth if staff are not clear on a patient’s response to the ethnicity question, then staff “ *normally will get where they were born”.* This could be a source of error as this interpretation may not reflect how the patient self-identifies. Participants wanted more advice on what to do in these situations:

*“I think maybe some guidance, like when you're unsure and then you have to try and guess yourself like, oh, they're from this area and this country or whatever.”*

Additionally, further sources of bias in GP data were identified where it was thought that third party identification may occur more frequently for certain ethnic groups.

*“I imagine there's a bias in not asking people who look manifestly White British…probably people’s instinct is to be more likely to ask when someone appears not to be”*

For HES, participants indicated that staff may feel reluctant to ask questions about ethnicity because of the view that it is *“awkward”, “sensitive”* and that *“it's something that, potentially, people can be offended by”*. Participants discussed that when asking *“sensitive questions”* relating to ethnicity, staff *“may use ‘not stated’ or ‘unknown’… instead of asking”.* This could potentially affect data completeness.

In contrast, for IAPT, participants did not raise specific concerns about the sensitivity of staff collecting ethnicity information from patients, although there was some discussion about variation of data collection between different practices. There was general understanding that ethnicity be self-identified by the service user regardless of how the data are recorded and by whom.

Variations in staff understanding and use of Not stated / Not known residual categories

For both HES and IAPT, participants raised concerns about misunderstanding and the inconsistent use of the ‘Not stated’ and ‘Not known’ categories.

‘Not stated’ is used to identify people who refuse to disclose their ethnicity and ‘Not known’ captures other reasons for missing information, such as a patient not being asked or being unable to answer. Distinguishing between these categories accurately has important implications for data quality and completeness, as a refusal is a valid response, whereas a ‘Not known’ should be followed up.

*“the trouble is we get… ‘not stated’ put in when the patient hasn't been asked… So, we have been trying to encourage our outpatient reception team to not only try and capture the data where it's unknown... but also to check where it's ‘not stated’”*

For GP, participants did not explicitly mention concerns around the ‘Not stated’ and ‘Not known’ categories, but did report there is no formal process for dealing with blank ethnicity records, and that practices may take different approaches. If a patient refuses to provide their ethnicity, staff may discuss with the patient why it is being collected. However this was dependent on the staff member and their understanding of why ethnicity information is collected.

In HES data, participants explained that the collection of ethnicity data is mostly treated as mandatory. However, the ethnicity variable is listed as required rather than mandatory, meaning that any records with missing required variables are not automatically rejected.

This finding that ethnicity is assumed to be mandatory, alongside the inconsistencies of how residual categories are used, raises questions around how missingness is dealt with during data collection. Further exploration around the use of ‘Not Known’, ‘Not Stated’ and ‘Other’ categories during data collection would likely be beneficial for understanding data quality.

***Data processing***

Data coding is a complex process for some data sources

Following data collection, the data must be processed into the appropriate format for submission to NHS Digital. For some data sources this can include converting written descriptions of ethnicity categories displayed during data input to numerical codes.

For HES, participants described how there is no possibility of inputting an incorrect code during data processing because you cannot deviate from the list of codes provided on the system.

However, for GP and IAPT, the coding process could be subjective and therefore inconsistent, and this was raised as a potential source of inaccuracy. For GP specifically, it was not clear whether ethnicity categories on registration forms would always match the options available on the IT system, particularly if bespoke categories have been added locally. Free text responses collected at GP surgeries must also be matched to a code which was reported as subjective. Participants noted that lack of guidance can lead to inconsistencies, and potential misrepresentation of certain ethnic groups.

*“…for example, Roma… may have been recorded as other European. Yeah, so it's not consistent…. within those numbers there will be some patients that haven't been recorded in the same way.”*

Mapping bespoke categories to Census categories has implications for data accuracy

Participants explained that for national level analysis, all data collected using bespoke local categories must be mapped to the standard Census categories.

For GP, participants highlighted that data mapping appeared to be a highly subjective process, with implications for data accuracy, particularly around over-coding of the 'Other' category. Comments indicated that at the start of the pandemic, a ‘ *rigorous*’ process was used to create the mapping for GP data once submitted to NHS Digital. Participants were not aware of any written report on this process, but recognised potential for error:

*“A sort of mapping eyeball exercise with some peer review…It wasn't totally dark, but there wasn't like a deterministic process that we could follow…So there's probably stuff that's got stuffed into ‘other’ which might be able to fit under a more specific group, but we didn't have the information to agree on it.”*

For IAPT, where data are mapped prior to submission to NHS Digital, participants flagged potential for inconsistencies across IT systems regarding which overarching category patients are coded to.

In contrast, for HES, whilst it was acknowledged mapping bespoke categories to Census categories resulted in a loss of granularity, there wasn’t the same emphasis on subjectivity. Participants interviewed about HES data collection described IT systems that use a nested approach in which each detailed category is automatically associated with a Census category. However, it was not clear if patients picking a more detailed ethnicity code in HES were aware of the Census code associated with the option they had picked and if not, whether that would be how they would self-identify.

Further exploration of the mapping process, particularly in relation to use of the ‘Other’ category could be valuable.

***Quality checks***

Role of NHS Digital and service providers in data quality

NHS Digital’s role on data quality was understood as *enabling*, meaning that they give service providers the tools required to make quality data submissions, but could not necessarily take action if incorrect data was submitted. For example in IAPT data collection NHS-Digital would not have the means to amend any data submitted incorrectly. 

Awareness of quality and quality assurance processes vary

Quality checks conducted at service provider level are not specified by NHS Digital. Consequently there were differences across health settings in both the checks completed by service providers to determine quality, and the level of overall engagement with quality assurance processes. This also varied between service providers of the same data source.

In IAPT it appeared that some service providers conducted their own checks to pick up quality issues and highlight them to “ *the therapist or the care coordinator”,* before monthly data submissions. However it was also highlighted that a small percentage of service providers do not submit at the overall high standard level, a group who were believed to be the least engaged and hardest to improve. This could be concerning particularly as limited measures are available to enforce data standards.

For GP and HES data, data submission by service providers was understood as a *“straightforward”* process. For HES data specifically, the fixed structure of the IT system meant that validation checks were not always conducted by service providers:

*“No, it's impossible to put a wrong code in 'cause the system…it has a pick list, so you can't deviate from that pick list…Uh, and in terms of validation we can't do any more validation on it because we don't have the patience to check”*

This raises the question of whether service providers are likely to validate the data they are inputting. Unlike other health data sources, it was also highlighted that invalid or missing ethnicity codes do not result in rejection or warning messages. When HES data is rejected the service provider does not receive any detail on which items in the data failed. This raises the question of how missingness is dealt with.

Data from GP surgeries are submitted by system suppliers on their behalf. Although using the IT system was considered straightforward by participants, understanding and interpreting how to match to ethnicity codes on the system was a source of confusion. Consequently it was unclear how accuracy could be checked at service provider level:

*“It is quite confusing to know which ones we should use. There seems to be a lack of guidance and a great degree of latitude in how each practice chooses to access the available options, how to interpret them and how to implement them…there’s still no clear-cut way of verifying if what you are doing is right”*

Participants described how service providers are engaged with the need for quality assurance and have tried to implement their own methods. For example, templates are used to “ *help standardise how different members of staff behave*” in GP surgeries although these weren’t entirely comprehensive.

It is challenging for NHS Digital to validate and quality check data already collected

When asked how data quality is checked by NHS Digital, participants highlighted the “ *trade-off"* between ensuring complete, high-quality data and reducing the data collection burden. Managing this means that NHS Digital aimed to make compromises with service providers to maximise submission of patient files. For example, when HES data are submitted records will be accepted with missing ethnicity information. Checks on records were described as labour intensive, therefore it was implied that ethnicity could receive less attention than high priority, mandatory variables such as NHS number or date of birth.

*'the problem is the report is sometimes thousands of lines long and it's knowing which ones to do? And I imagine ethnicity is probably not one they jumped to straight away.*

For HES, participants described some automated validation checks when data are submitted, though these mainly focused on checking the file structure is valid rather than the file content. NHS Digital checks generally focus on completeness rather than accuracy, which is more difficult and time-consuming to check. For example in HES, cleaning and validating “unknown” or “blank” variables was the priority.

Overall, participants noted “ *the bar for validation is set fairly low and only on certain very…key, high profile data items*”. This highlights the importance of the role of service providers in ensuring data quality prior to submission to NHS Digital.

Additional checks are not required by NHS Digital and raises the question of consistency of quality across service providers, particularly around missed data from those providers not conducting additional checks.

NHS Digital central initiatives focus on data completeness

NHS Digital aims to encourage service providers to take responsibility for data quality. To help strengthen this sense of responsibility and encourage improvements, NHS Digital have developed various tools to improve service providers’ understanding of the data quality.

I.   Quality reports

Examples mentioned included reports automatically generated when IAPT data is submitted, allowing data submitters to review rejections and warnings. Additionally, the NHS Data Quality Maturity Index was referenced as a monthly publication, which again provides information on data quality including the ethnicity variable, and a scoring.

For IAPT, participants did not mention any financial incentives for service providers improving the quality of data submitted to achieve a higher scoring in these reports. This suggests that NHS Digital rely on the motivation of service providers to act on any quality issues flagged in these reports.

Furthermore, as with any target, it is important to be aware of potential unintended consequences. For example, participants felt that there could be a valid reason for recording ‘not known’, particularly in hospital settings.

*“you could argue … a patient that comes in unconscious and therefore…it might be a genuine reason to record that.”*

However, in NHS Digital’s Data Quality Maturity Index Scoring, the use of the ‘not known’ category is often highlighted as a ‘meaningless’ code which can “ *drag provider scores down*”. This could have negative unintended consequences if it inadvertently encourages the use of ‘‘not stated’ or ‘other’ rather than ‘not known’.

II.   Financial incentives

Participants mentioned financial incentive schemes for GP data collection. While these were generally not specific to ethnicity, we found evidence that GP surgeries can be motivated by financial incentive schemes.

*“It seems that all we're doing over the last couple of years is trying to find ways of generating money into the practice…we try and go for it because we desperately need the money”*

However, we also found evidence that GP surgeries have previously found it confusing to implement changes and receive financial benefits that do relate to ethnicity, because of the long list of both legacy and up-to-date ethnicity codes included on their IT system. Despite code descriptions being similar, only some codes resulted in requirements for funding being met. GP practices were unclear on what codes to choose to meet incentives, and guidance was limited.

Incentives focused on improving data completeness rather than accuracy. With concern around funding being evident, it raises the question of whether selecting certain codes to meet incentives could bias the data being collected.

For IAPT no current financial incentives were mentioned although they were raised as a potential way to motivate service providers to improve data quality. Ethnicity data was considered part of “ *key performance indicators*” within the NHS and this could be developed to increase motivation to collect good quality data.

Participants for HES did not mention specific financial incentives relating to ethnicity data collection.

***Training and guidance***

The usefulness of guidance, training and support on ethnicity data collection varies between sources

Participants’ views on the usefulness of guidance and training on collecting ethnicity data varied greatly between different sources.

For GP data, participants described guidance as limited and the guidance that *is* received was not considered helpful or easily understood:

*“There is guidance. The problem is trying to identify kind of the authoritative guidance, and which is the right source…there's a lack of clarity about what we are really meant to be doing, and some of the guidance we get will be so…dense and contained in such a big government document that it's hard to read”*

Lack of guidance raises questions about the impact not only on data quality but relationships between service providers and NHS England. For example, it was described that some Primary Care Networks (PCNs) were unable to deliver contract requirements regarding data collection, and this was implicitly linked to a lack of guidance:

*“We have to be empathic to the situation, but at the same time it's a contract. So, it's a really challenging place to be. We receive complaints from the PCNs.”*

In General Practice Extraction Service (GPES) IT systems, ethnicity information is stored using SNOMED CT health terminology. NHS Digital do provide some ‘Implementation Support’ to GPs on how SNOMED codes should be used. However rather than contacting NHS Digital, GP surgeries found it more practical to seek support from colleagues and the PCN.

There was agreement that improved guidance for ethnicity data collection is needed, but comments were made about “ *red tape*” which made guidance difficult to develop. Specifically, participants explained how ongoing challenges with the COVID-19 pandemic meant priorities changed, and detailed guidance that was developed through engagement with clinicians and other health professions was heavily reduced.

*“…we've provided recently…a fuller guidance, but there were some challenges…that it was too detailed, and within that initial guidance, we've put a lot of detail in about how to capture this information. However, we've had to minimize that sadly”*

Participants were most positive about the guidance for IAPT, the newest of the three data sources. Participants indicated it was clear what NHS Digital required and guidance is accessible on the NHS Digital website and via the IAPT manual. 

For HES, participants were broadly aware of data standards required, and there was little demand for further guidance or training. However, there was evidence of issues that could be prevented with better guidance. These focused on the role of staff in data collection and included perceptions of ethnicity as a static characteristic where self-identification cannot change, the perception that data quality is guaranteed by the coding systems, and reluctance to ask a patient about their ethnicity.

Staff understanding of the value of collecting ethnicity information varied across data sources

For IAPT and GP data, participants understood the value of collecting ethnicity data and considered it useful for funding, understanding populations, addressing potential needs of populations and monitoring inequalities. 

In particular, it was explained how improving IAPT data on protected characteristics can, and has, led to more tailored practices and treatments for such groups. There was evidence that staff understanding of the value of ethnicity information may influence both their ability and efforts to ensure data completeness.

For HES, participants indicated that ethnicity data collection for staff at Trust level is not assumed to be *“at the top of [the] list of things to make sure they’ve got recorded*”. Hospitals are a more challenging environment in which to collect ethnicity data, as providing urgent medical care must take priority over other tasks such as ethnicity data collection. Participants made a distinction however between the “ *stressful*” Emergency Department and the “ *calmer*” outpatient setting which is an “ *easier environment*” to capture ethnicity in. Participants also mentioned how organisational culture can affect the value staff place on collecting and ensuring the quality of ethnicity information.

***Patient opt out from data sharing***

Limited information is available on those opting out of data sharing

Participants explained that patients can choose to opt out of sharing their patient information. There is limited information on those who choose to opt-out, so it is difficult to understand or account for bias in coverage of ethnicity as a result of the opt-out process.

## Conclusions/Discussion

### Summary of main findings

Previous research undertaken by the Office for National Statistics compared ethnicity information recorded in hospital records and a subset of GP records to ethnicity information recorded by the same person in their 2011 Census response (Census data is self-reported so widely considered the most robust ethnicity source for the whole population). This research found that:

- For both GP and hospital data sources, agreement with 2011 Census was highest for the White British category (greater than 96%), with South Asian, which includes Bangladeshi (greater than 91%), Pakistani (greater than 86%) and Indian (greater than 81%), and Chinese (greater than 81%) categories also reporting high agreement rates.
- However, for both GP and hospital sources, agreement was lower for all Mixed ethnic groups (less than 67%) and other ethnic groups, including Other Asian (less than 60%), Other White (less than 55%), Other Mixed (less than 21%), Other Black (less than 16%) and Any Other ethnic group (less than 15%).
- For most ethnic groups, agreement rates for hospital records were lower compared to agreement rates for the GP records included in the analysis.

These differences in the accuracy of recording are likely to impact estimates of ethnic health disparities. Whilst there are set standards for data submissions to NHS Digital, there are clear deficiencies which can result in bias and inaccuracies in the data recorded, and subsequently used for planning, operational, and research purposes.

This qualitative analysis of ethnicity-specific data collection processes has identified numerous points at which errors and assumptions can permeate individuals’ health records. For example, although participants interviewed in this study were aware of NHS Digital data standards, some believed that they had freedom at the local level, and could act flexibly to meet requirements. The subjectivity that then exists in the data collection process means that individual staff collecting and coding ethnicity data could conceivably record ethnicity information for the same person differently.

Below we reflect on three key types of barriers to effective and accurate recording of ethnicity data in routinely collected health records: 1) data infrastructure challenges; 2) human factors challenges; and 3) institutional challenges. Specifically, as we summarise these, we have sought to correlate the findings of this study with the literature-at-large, and where possible identify practical solutions.

***Data Infrastructure***

A key theme that emerged from the qualitative interviews is that there are several ontologies for recording routinely collected health data, including ethnicity, and these are often capable of accommodating different degrees of granularity. This has also been described previously in the literature, wherein Morrison *et al*. (2014) highlight that GP IT systems allow more detailed ethnicity categories to be entered than the standard categories based on the 2001 Census.

Although Morrison *et al*. (2014) did not find much evidence of variation in ethnicity categories used in hospitals, our research found evidence of varied detailed categories being used for data collection in all three data sources in scope of the research. It seems that the use of detailed ethnicity categories has not only persisted, but it is possible that the range of available options may have expanded; previously Morrison *et al.* (2014) identified 83 Read codes available for ethnicity entry in GP IT systems, compared to the 489 SNOMED codes available for ethnicity entry we have identified as of April 2022. Consistent with our findings, the 83 Read codes identified by Morrison *et al.* also appear to conflate religion and country of birth with ethnicity.

The breadth of ethnicity categories available at the point of data entry compared with the standard Census codes that are subsequently used by analysts means that such granularity is largely reduced throughout the data collection process. Service providers may collect data at the granular level for the purposes of serving local population health needs, and yet this does not correspond with the wider purpose of data collection by NHS Digital, whereby aggregations are useful for analysis at the national level. This disconnect of ontologies, and their purposes, has a meaningful impact on service providers’ ability to record information accurately and effectively.

A solution to this could be the introduction of clear mapping of ethnicity codes that is implemented across health data sources, whereby a hierarchical structure is produced to ensure detailed ethnicity categories can be aggregated using a standardised process. To ensure the data collection process meets the needs of both analysts working with high level Census categories and service providers adapting to local health needs, it is acknowledged that such a system would need to be dynamic. This means that the hierarchical structure would have flexibility to adapt to different codes being collected by specific service provider, yet can still be mapped to aggregate ethnicity categories in systematic way, regardless of which data source is being used.

The use of detailed categories within the current data infrastructure has implications for analysts. The finding around the possible over coding of local categories to ‘Other’ supports the use of methods to re-allocate the ‘Other’ category to reduce the risk of undercounting ethnic minority groups. There could also be opportunities for further research to analyse the detailed local codes collected to help improve accuracy, and potentially help produce an improved, hierarchical mapping system.

***Human factors***

The challenges of human behaviour on the collection of ethnicity data can be summarised three-fold. Firstly, how members of the public self-identify and their understanding of the ethnicity classification system may not be consistent with the categories presented to them in health settings.

Public understanding of ethnicity classifications is compounded by the second challenge of the interaction between service users and service providers when collecting patient information. Previous research has shown that staff training is a key factor affecting data quality, and that multiple barriers exist to ethnicity data collection within hospital settings: collecting data from patients was seen as time consuming with implications for resourcing; the clinical relevance of the data was not always known by NHS staff, and fear of causing offence to the patient were all cited as barriers to completing the ethnicity data item. ( Iqbal *et al*., 2012: 286-287; Morrison *et al*., 2014).

Our qualitative analysis supports this literature where the role of staff in each health setting was reported to affect how data are collected. For example, participants reported that third-party completion of GP registration forms meant the possibility of bias through subjective interpretation, and in particular hospital settings such as A&E, ethnicity data are sometimes not collected at all due to clinical priorities.

Qualitative interviews also found that variations in training and guidance can affect capability and confidence of staff collecting data from service users, linking to existing literature on how staff training affects data quality ( Iqbal *et al*., 2012: 286-287; Morrison *et al*., 2014). Differences were identified across the three data sources between: staff concerns over a lack of guidance and actively seeking more help; staff perceptions around the simplicity of data collection meaning that there is no desire for more guidance; and adequate training and guidance provided and staff being content with this. Specifically, a lack of clear guidance in GP data was reported to affect service providers’ ability to collect accurate data and meet financial incentives, which has an impact on relationships with NHS Digital. Developing improved guidance for staff was considered challenging. In addition, varied understanding and use of residual categories were reported, which presents another potential bias at the point of interaction between patient and staff. 

The third barrier identified was how the data collected are then coded by staff ready for submission to NHS Digital, which could affect accuracy. Findings revealed that for some data sources, mapping bespoke ethnicity categories was considered complex, particularly if the categories on the data collection form do not match the IT system. Patients may also not be aware of how their data entry could be aggregated, and it was reported that complexities of the process could result in the over-coding of ‘Other’. 

These layers of variation in human behaviour threaded through the data collection process contributes to the potential for incorrect information being collected, and assumptions made by individuals to accommodate the rigid ontologies specified by NHS Digital. This issue of human influence on data collection could be improved by the development of adequate public engagement on both the importance of collecting ethnicity data and the classifications that are used.

Importantly, there is a need for detailed guidance and training to cover each stage of the data collection process. The development and implementation of such guidance would help contribute to both a standardised data infrastructure aforementioned, as well as a standardised method of data collection. This standardised method, supported by such guidance would ideally account for and alleviate the specific challenges that service providers face in their particular health settings, that currently hinder accurate data collection.

Guidance could also be extended beyond service providers to analysts working with these health data sources. For example, based on our findings analysts would need to be aware that, in their current state, exclusion of Other, Not stated, Unknown from analyses of these data sources would likely to result in undercounting of ethnic minority groups.

There are also ethical considerations associated with how analysts handle the Not Stated category, especially where analysts can view multiple records for one patient where the most recent response is a refusal, but previous records contain a stated ethnicity code. Further research would be needed to establish whether a Not Stated response always equates to a refusal, or whether patients who are not being asked are also sometimes coded as Not Stated.

***Institutional factors***

The final theme identified from qualitative interviews relates to the finding that centralised initiatives and incentive schemes have a clear focus on data completeness, that inadvertently impacts the accuracy of ethnicity data. 

Existing research suggests past central initiatives to improve quality of ethnicity recording in GP data and some HES data sources have been effective in substantially improving data completeness ( Mathur *et al*., 2014). However, research conducted since these improvements has raised concerns about the accuracy of ethnicity information recorded. When ethnicity data from the HES inpatients dataset was compared with national population estimates by age group, this showed that ‘…in most cases hospital records over-represent ‘other’ categories while under-representing ‘mixed’ ethnic groups, and some specific ethnic groups.’ Similarly when ethnicity recorded in HES and GP data were compared to 2011 Census, high levels of disagreement were reported for ‘Mixed’ and ‘Other’ ethnic groups. 

The Data Quality Maturity Index dashboards available on the NHS Digital website present metrics of various data quality dimensions for HES and IAPT: coverage, consistency, completeness, validity and accuracy. The data quality information for IAPT also provides an evidence-based assessment of the quality of the dataset by reporting against those of the European Statistical System (ESS) quality and related dimensions and principles, however due to the nature of the data collection and challenges around assessing data accuracy, the main evidence available for assessing data accuracy are tables showing the proportion of Valid, Other, Default, Invalid, Missing codes.

This evidence is reflected in findings from qualitative interviews. Participants’ accounts of NHS Digital quality assurance processes, quality reports and financial incentives around data collection all imply that data completeness is a priority across all three data sources. A clear example of this was the finding that for HES, quality checks focused on checking the file structure is valid rather than the file content. This approach is grounded in the complex balance of ensuring complete, accurate data versus reducing data collection burden on service providers.

Potential solutions are therefore linked to this issue of completeness versus accuracy; with the latter being difficult to achieve because ensuring accuracy requires verification of ethnicity by the individual. In principle, by implementing the two solutions suggested for improving data infrastructure and reducing bias from human factors, data accuracy would be indirectly addressed. Other methods could also be considered based on novel data linkages or supplements.

Participants also mentioned future developments being explored that may impact ethnicity data collection going forward. For example, updating the ethnicity categories from the 2001 Census categories to the Census 2021 categories.

Key sources of error and bias identified from analysis are summarised in Figure 1.

### Implications for analysts

Issues of potential bias and error highlighted from this qualitative research reinforce the importance of analysts following good practice when handling ethnicity in health datasets.

Analysts must ensure that they: assess data quality thoroughly and cross-validate against similar data sources; provide evidence of how any miscoding has been addressed in their analysis; communicate caveats in any reports; interpret results with caution; and consider caveats when drawing any conclusions or recommendations. Finally, any future improvements to data collection processes mentioned above would need to be considered when conducting any time series analysis. The point at which data collection methods change could impact comparisons over time, as data for previous years would be based on the existing system.

### Strengths and limitations

***Strengths***

This study sought to collect data on the collection of ethnicity information for three health related data sources, as well as the experiences of those involved in the end-to-end process. By using qualitative methods, we looked to uncover the real-world circumstances surrounding ethnicity data collection, and answer any gaps in knowledge following the literature review. By employing purposive sampling, we sought to ensure relevant data collection, as well as looking to collect a range of experiences from those in different roles. Semi-structured interviews afforded the opportunity to structure conversations around our research aims whilst also giving the flexibility for participants to relay individual experiences. Topic guides were informed by the literature review and were peer-reviewed and piloted to ensure research aims were adequately addressed. In addition, we ensured that interviews were attended by two researchers, one of whom took notes, as well as recording and transcribing the interviews sessions with the view to minimise any errors in our data collection. The analysis was collaborative, reflective, and peer-reviewed at all stages.

***Limitations***

Despite measures, the study did have some limitations. The sample size was small and, whilst saturation did appear to be achieved as a whole, one data source did suffer from a smaller sample than the others (GDPPR). Additionally, despite purposive sampling measures, there were some parts of the end-to-end process that participants did not have knowledge on and thus could not provide us with detail on. This results in some outstanding knowledge gaps yet to be informed. The qualitative study represents the thoughts, views, and experiences of our sample, and is not designed to be representative. Our findings are also limited to the scope of the research and wider circumstances. This means that our findings are limited to the experiences of those employed within a health setting, as opposed to those using health services. In addition, it is acknowledged that a change in wider circumstances, for example moving to 2021 Census ethnicity categories, may change the relevance of the findings.

## Data Availability

Transcripts of interviews with participants as part of this study are not to be shared due to ethical restrictions regarding anonymity and confidentiality. Participants were selected for their knowledge and experience of different stages of the NHS data collection process. Due to the specialist roles undertaken by many of the staff interviewed, there is a risk that participants could be identified from audio recordings and transcripts of participant interviews, which would compromise the privacy of our research participants. Instead, detailed findings and quotations relating to each individual data source (prior to synthesis for the final report) have been anonymised and made available in the Extended Data section. Those wanting to review the data underlying this research should see the Extended Data, which has been designed to allow detailed findings from the interviews to be shared, whilst protecting the privacy of participants. Figshare: Summary of key findings from interviews relating to each of the data sources included in the study ‘Methods and systems used to collect ethnicity information in health administrative data sources in England'. https://doi.org/10.6084/m9.figshare.22325203 ( Quayle *et al.*, 2023) This project contains the following extended data: Extended_data_for_upload.docx (Summary of key findings from interviews relating to each of the data sources included in the study ‘Qualitative interviews to understand methods and systems used to collect ethnicity information in health administrative data sources in England’.) Figshare: SRQR checklist for ‘Qualitative interviews to understand methods and systems used to collect ethnicity information in health administrative data sources in England. https://doi.org/10.6084/m9.figshare.22325578 ( Drummond & Tabor, 2023) Data are available under the terms of the Creative Commons Attribution 4.0 International license (CC-BY 4.0).

## References

[ref-1] Drummond R Tabor D : SRQR - "Qualitative interviews to understand methods and systems used to collect ethnicity information in health administrative data sources in England".figshare. Online resource,2023. 10.6084/m9.figshare.22325578.v1 PMC10521056 37766853

[ref-2] Iqbal G Johnson MRD Szczepura A : Ethnicity data collection in the UK: The healthcare professional's perspective. *Divers Equal Health Care.* 2012;9(4):281–290. Reference Source

[ref-3] Morrison Z Fernando B Kalra D : The collection and utilisation of patient ethnicity data in general practices and hospitals in the United Kingdom: a qualitative case study. *Inform Prim Care.* 2014;21(3):118–31. 10.14236/jhi.v21i3.63 25207615

[ref-4] Mathur R Bhaskaran K Chaturvedi N : Completeness and usability of ethnicity data in UK-based primary care and hospital databases. *J Public Health (Oxf).* 2014;36(4):684–92. 10.1093/pubmed/fdt116 24323951 PMC4245896

[ref-5] Quayle G Jones B Atkins J : Summary of key findings from interviews relating to each of the data sources included in the study ‘Methods and systems used to collect ethnicity information in health administrative data sources in England'. figshare. [Dataset],2023. 10.6084/m9.figshare.22325203.v1 PMC10521056 37766853

