# Supplementary material for: Qualitative interviews to understand methods and systems used to collect ethnicity information in health administrative data sources in England
Source: Wellcome Open Res. 2023 Jun 21;8:265. [Version 1] doi: 10.12688/wellcomeopenres.19262.1 (PMC10521056; doi:10.12688/wellcomeopenres.19262.1)
Supplement: Peer review history

# Reviewer report: Reviewer response for version 1

**DOI:** 10.21956/wellcomeopenres.21345.r60877

**Published:** 2023-09-15

**Authors:** Gulnaz Iqbal

**Affiliations:** 1 Warwick Clinical Trials Unit, University of Warwick, Coventry, England, UK

**Introduction**

 This is an interesting paper in an area where little research has been conducted. The completeness of ethnicity data collection is improving but data accuracy remains a problem.

**Methods**

 The methods of the literature review are not provided.

 Please include any databases searched, search terms, and timeframe of the literature and grey literature used to inform the research questions.

 A copy of the topic guide used for the focus groups should be included. The authors do not mention who the topic guides were peer reviewed by or piloted on.  

**Results**

 Results from the search of the literature should be broadly summarised and/or listed in the references. Findings from the focus groups are well structured but it would be helpful to see demographics information of the sample e.g. age, sex and ethnicity.  

**Conclusions/Discussion**

 Strengths and limitations are well described for this small study and it adds to the knowledge base.

Is the work clearly and accurately presented and does it cite the current literature?

Yes

If applicable, is the statistical analysis and its interpretation appropriate?

Not applicable

Are all the source data underlying the results available to ensure full reproducibility?

No source data required

Is the study design appropriate and is the work technically sound?

Yes

Are the conclusions drawn adequately supported by the results?

Yes

Are sufficient details of methods and analysis provided to allow replication by others?

Partly

Reviewer Expertise:

Statistician with some experience in qualitative research. Expertise in ethnicity data collection in healthcare.

I confirm that I have read this submission and believe that I have an appropriate level of expertise to confirm that it is of an acceptable scientific standard, however I have significant reservations, as outlined above.

# Reviewer report: Reviewer response for version 1

**DOI:** 10.21956/wellcomeopenres.21345.r66693

**Published:** 2023-09-15

**Authors:** Sarah Masefield

**Affiliations:** 1 Department of Health Sciences, University of York, York, England, UK

This paper provides a valuable qualitative insight into potential issues and inadequacies in the reporting and recording of ethnicity data in health administrative data sources in England. The authors clearly outline many potential explanations for the uncertainties those of us working with administrative datasets frequently encounter in conducting analyses and interpreting the data/findings. The research is thorough, and the methods are appropriate. Thank you for this elucidatory and well presented research.

 This paper presents research that has been available on the ONS website since January 2023. The presentation of the research in more detail as a research paper will help it reach a wider/different audience but for transparency, it would be appropriate for the link between the two to be made clear in the text of the research paper (not only through a hyperlink on Figure 1).

 Please provide a bit more information on why you selected for the GPES, GDPPR, HES and IAPT datasets. Do you expect commonalities between the findings for these and other health administrative datasets (e.g. maternity or mental health services)? To what extent are the differences observed between ethnicity data collection and entry in the three different datasets likely to be common to all administrative datasets e.g. is ethnicity data treated as mandatory or required in most datasets, is open text or closed (i.e. drop down list) coding for ethnicity most commonly used in administrative datasets in England? Are there potential limitations for the application of your recommendations to other administrative datasets?

 I would like to see a bit more detail on how the framework used for the analysis was generated (by whom, based on what knowledge/expertise) and how it relates to the findings – there is a little about this in the strengths section but please include more in the methods. Was this therefore a framework analysis? The authors say the framework is linked to the structure of the topic guide but in that case, how was this structure developed? Does the topic guide map onto the elements of Figure 1? If this is to some extent, please tell us which elements came from the organising framework and which were emergent themes from the analysis. The framework is mentioned in the methods but absent from the discussion. I’d like to see this addressed.

 It would have been interesting to have more depth on the bespoke ethnicity recording occurring in local contexts (beyond the 16+1 categories). Were any examples provided? What is the impact on the NHS system i.e. is there a separate layer of local administrative data recording that is superseding the NHS Digital requirements, or is there double recording so that the 16+1 codes are used as well as the local categories and if so is the outcome a collapsing of the data into the 16+1 categories or is it introducing missingness or inaccuracies? I think this is touched on but could perhaps be made more explicit.  

 An additional recommendation could be that where not currently included in the metadata for these datasets, it would be useful for summaries of the limitations of the ethnicity data in each dataset to be included to inform and ensure their cautious interpretation.

 Thank you for including the extended data.

Is the work clearly and accurately presented and does it cite the current literature?

Yes

If applicable, is the statistical analysis and its interpretation appropriate?

Not applicable

Are all the source data underlying the results available to ensure full reproducibility?

Yes

Is the study design appropriate and is the work technically sound?

Yes

Are the conclusions drawn adequately supported by the results?

Yes

Are sufficient details of methods and analysis provided to allow replication by others?

Yes

Reviewer Expertise:

Health adminstrative data; health inequalities; perinatal mental health; parent and child health

I confirm that I have read this submission and believe that I have an appropriate level of expertise to confirm that it is of an acceptable scientific standard.

# Reviewer report: Reviewer response for version 1

**DOI:** 10.21956/wellcomeopenres.21345.r63085

**Published:** 2023-09-15

**Authors:** Suman Prinjha, Ash Routen

**Affiliations:** 1 Population Health Sciences, University of Leicester, Leicester, England, UK

This is a clearly written manuscript that focuses on an important topic: how ethnicity data are collected, the quality of these data, and potential sources of error and bias in the process of collecting NHS ethnicity information. Three key NHS data sources that cover primary and secondary care are discussed – General Practice Extraction Service for Pandemic Planning and Research (GPES, GDPPR), Hospital Episode Statistics (HES), and Improving Access to Psychological Therapies (IAPT). Twenty-two semi-structured qualitative interviews were conducted with experts working on different aspects of health admin data collection to explore potential sources of bias when collecting ethnicity information. Three key sources of error and bias were identified across data collection, data processing, and quality assurance processes. Three main barriers that can result in bias and inaccuracies were discussed: data infrastructure challenges, human challenges, and institutional challenges.

 The authors suggest potential solutions. The manuscript highlights that analysts using health admin data should be aware of the main sources of potential error and bias in health admin data, which are more likely to affect the ethnicity data for ethnic minority groups, and seek to account for this bias in their research. The study is part of a wider research collaboration between ONS, Wellcome Trust, and the Race Equality Foundation to improve understanding of the quality of ethnicity data in key NHS sources, and develop methodological solutions to reduce biases.

 This is a well-written, interesting and important manuscript, which we enjoyed reading. More clarification on the following points would be helpful.

**1. Introduction**

 This section reads a little more like an internal report, rather than the background and justification for an academic paper. To rectify this, we would suggest the following:

 (i) The authors refer to issues with ethnicity data quality. What defines quality?  It would be helpful if the authors could briefly state specifically what this is? Likewise, when referring to inconsistencies? Is this in the completeness of the data? Accuracy? Stability over time? If there is space, some numbers or a statement of direction of inconsistency between different data sources would be useful.

 (ii) Provide citations in paragraph 2 and 3 to support the statements made.

 (iii) On introducing the concept of a desk review it would be useful to briefly define this methodology and provide a citation, as it is not widely used or understood. In addition, the authors move from describing some background literature/justification to announcing ‘this desk review’ which assumes that the reader has some prior knowledge of this project. 

 (iv) It is fine to introduce the wider project, but it needs to be woven into the narrative that justifies this work in the context of existing literature and evidence gaps.

 (v) Remove the final paragraph of the introduction referring to implications of the work. This should come in the Discussion section.

 (vi) Remove bullet points and rewrite as continuous prose to improve flow for reader.

**2. Sampling and recruitment:**

 (i) 22 participants were interviewed (21 interviews; 1 participant provided email information). Can any biographical information be included under participant quotes (e.g. gender, age)?

 (ii) Researchers designed and peer-reviewed topic guides which were piloted. What did this involve? Were any patients included in peer-reviewing? It would be helpful to include examples of the kinds of questions that were included in the topic guides or to include the topic guide as supplementary material.

 (iii) Interviews were conducted by a lead interviewer and a second researcher observed. Can the authors include in brackets the initials of the authors who conducted the interviews? For example:Interviews were conducted by a lead interviewer (XX) on Microsoft Teams and lasted between 40 to 80 minutes. A second researcher (YY) attended the interview as an observer to take notes of responses.

**3. Methods**

 (i) How was consent gained – written, verbal etc.?

 (ii)  How were participants recruited?  E.g., via word of mouth, social media, letters to organisations....?

 (iii) The data collection section is written as a series of discrete statements. To improve flow for the reader, we suggest rewriting as continuous prose.

 (vi) Who (include author initials) and how many people conducted the analysis?

 (v) Can the authors be more specific about the type of thematic analysis e.g. reflexive? It would also be useful to state that the approach was inductive.

**4. Results**

 The Results section provides a lot of helpful detail but is presented in a way that makes it confusing to read and slightly repetitive. Presenting a summary of the main findings first seems unnecessary, and does not appear to be based on the interview data. The Results would be easier to read if:

 (i) The summary of main findings was deleted from Page 4. Theme headings would be easier to read if they were numbered. For example:

**Results**

 Our research identified ways in which data quality could be affected at different stages of the data collection process by patients and service providers, shown in Figure 1.  This section presents more detailed findings from our research presented under the following themes: (1) Data Collection; (2) Data Processing; (3) Quality Checks; (4) Training and Guidance (5) Patient Opt Out from Data Sharing. 

***1. Data collection***

 1.1 Transfer of existing health records for data collection varied between sources [Add findings presented in manuscript]

 1.2 Variation in ethnicity categories collected locally [Add findings presented in manuscript]

 1.3 The mode of data collection could impact data completeness [Add findings presented in manuscript]

 1.4  Variation in how....etc using this format throughout the Results section  

***2. Data processing***  

 (ii) A table of key themes, with sub-themes below each heading, would be helpful.

**5. Conclusions / Discussion**

 Good discussion overall. The authors state that they will reflect on three key types of barriers to effective and accurate recording of ethnicity data in routinely collected health records. It would be helpful to number the 3 barriers in this section for ease of reading.

 In the ‘Data Infrastructure’ section, it would be helpful to discuss this as a barrier rather than ‘a key theme’. Similarly, in ‘Institutional factors’, it would be helpful to say ‘the final barrier’ rather than ‘theme’. The Abstract should be revised to reflect these changes.    

 The Discussion starts with a discussion of previous literature, while the heading ‘summary of main findings’ is used. Instead, this section should start with restating the aim of the study, and then summarise the headline findings in continuous prose. As above, we would suggest removing the bullet points.

 When referring to the NHS digital data standards, it would be useful to provide a citation for the reader to follow-up on.

 Please briefly define ontologies in this context, as we are unsure what the authors mean here.

 In the strengths sub-section the authors refer to a literature review – what review is this and can it be cited?

 In the limitations section the authors state ‘Despite measures’ – it is unclear what this means?

 The authors mention data saturation. How was saturation defined? It would be useful to mention data saturation in the methods section.

 What is an ‘end-to-end’ process?

 It would be useful to include a conclusions section or a brief concluding statement at the end of the Discussion.

**6. References**

 It may be useful to refer to the ongoing work of HDR UK on ethnicity data quality and coding, as well as previous work on improving ethnicity data recording and coding, and examinations of ethnicity coding quality in health datasets.

https://ukhealthdata.org/projects/diversity-in-data/

 Routen, A., Akbari, A., Banerjee, A. et al. Strategies to record and use ethnicity information in routine health data. Nat Med 28, 1338–1342 (2022). https://doi.org/10.1038/s41591-022-01842-y

 Scobie, S. et al. Ethnicity Coding in English Health Service Datasets (Nuffield Trust, 2021).

Is the work clearly and accurately presented and does it cite the current literature?

Partly

If applicable, is the statistical analysis and its interpretation appropriate?

Not applicable

Are all the source data underlying the results available to ensure full reproducibility?

No

Is the study design appropriate and is the work technically sound?

Yes

Are the conclusions drawn adequately supported by the results?

Yes

Are sufficient details of methods and analysis provided to allow replication by others?

Partly

Reviewer Expertise:

Qualitative research; ethnic minority health; health inequalities

We confirm that we have read this submission and believe that we have an appropriate level of expertise to confirm that it is of an acceptable scientific standard, however we have significant reservations, as outlined above.

## References

[1] : Strategies to record and use ethnicity information in routine health data. *Nat Med* .2022;28(7) : 10.1038/s41591-022-01842-y 1338-1342 10.1038/s41591-022-01842-y 35641824

[2] : Ethnicity Coding in English Health Service Datasets. *Nuffield Trust* .2021; Reference source
